# Supplementary material for: Phase biasing of a Josephson junction using Rashba–Edelstein effect
Source: Nat Commun. 2023 Nov 16;14:7415. doi: 10.1038/s41467-023-42987-9 (PMC10654735; doi:10.1038/s41467-023-42987-9)
Supplement: Supplementary file 1 — Supplementary Information [file 41467_2023_42987_MOESM1_ESM.pdf]

Supplementary information *for* Phase biasing of a Josephson junction using Rashba-Edelstein effect

Tapas Senapati[1], Ashwin Kumar K[2], Kartik Senapati[1*]

[1]School of Physical Sciences, National Institute of Science Education and Research (NISER) Bhubaneswar, An OCC of Homi Bhabha National Institute, Jatni, 752050, Odisha, India.
[2]Department of Physics, Birla Institute of Technology & Science Pilani - K K Birla Goa Campus, Zuarinagar, 403726, Goa, India.

Supplementary Note 1: Characterisation of deposited films

The platinum thin films were grown using magnetron sputtering with a rotating substrate holder plate to control the thickness. Although the Pt films grown on Si/SiO_2 were polycrystalline, a preferential orientation in the (111) direction was observed. The XRR plot for different thicknesses of Pt is shown in Supplementary Fig. 1(a), along with the respective fits. The thicknesses we got here were 12.7nm, 25nm and 50nm, with film roughness of less than 0.3nm. Supplementary Fig. 1(b) shows the $\theta - 2\theta$ X-ray diffraction pattern for a multilayered film Pt(30nm)/Cu(20nm)/Nb(3nm). The Nb layer, in this case, was grown to avoid oxidation of the Cu layer with the $\mathrm{SiO_2}$ surface, which degrades the adhesion of the Cu layer on $\mathrm{SiO_2}$ substrate. The XRD pattern shows that both Cu and Pt layers have a preferential (111) growth direction, though polycrystalline in nature. It has been shown in the literature that the (111) face of Cu is most suitable for observation of the Rashba-Edelstein effect.

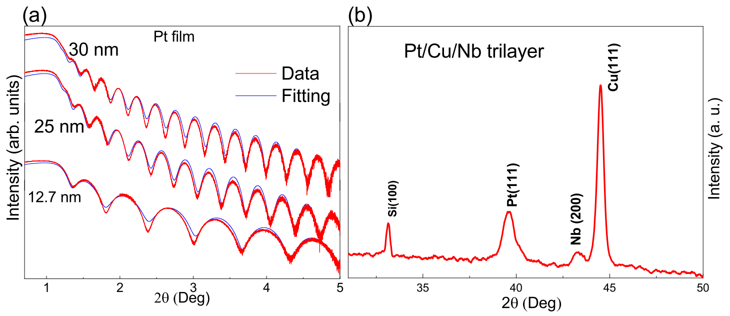

Supplementary Fig. 1: Characterisation of Pt-thickness and Pt/Cu multi-layered structure: (a)X-ray reflectivity measurements performed on Pt thin films to estimate Pt- thickness. The blue lines are fitted curves to the experimental data.**(b)**X-ray diffraction pattern for a Pt(30nm)Cu(20nm)Nb(3nm) multilayered film deposited on Si/SiO_2. This data shows that both Pt and Cu layers grow in a preferentially (111) direction, although they are polycrystalline. The Nb-layer, in this case, was used as a capping layer to prevent oxidation of Cu.

Supplementary note 2: Transport properties of a Nb-(Pt/Cu)-Nb planar Josephson junction

In Supplementary Figure 2, we show the essential characteristics of the planar Nb-(Cu/Pt)-Nb junctions with 25nm Pt layer. These general features were consistently obtained in all the fabricated junctions with Pt layer thickness varying from 10nm to 50nm. As shown in Supplementary Fig. 2(a), the junctions show a two-step superconducting transition. The transition at higher temperatures ($\sim$ 7.6 K) corresponds to the onset of superconductivity in the Nb electrodes of the junction. In comparison, the broader transition at the lower temperatures ($\sim$ 4.5 K in Supplementary Fig. 2(a)) corresponds to the onset of Josephson coupling across the Nb electrodes. The proximatization of the Cu layer in the barrier realizes Josephson coupling in these planar junctions. The current-voltage (IV) curve for the same junction measured at 2K is shown in the inset of Supplementary Fig. 2(a). A critical current of $\sim$ 650μAmps was observed in this case. A definitive test of Josephson coupling across superconducting banks in a device is the typical Fraunhofer-like variation of critical current as a function of the magnetic field. Equivalently, the voltage of a Josephson coupled junction at a fixed bias current, close to the critical current, also follows a similar but inverted field dependence. In Supplementary Fig. 2(b), we compare the magnetic field dependence of critical current and junction voltage for the Nb-(Cu/Pt)-Nb junction with a 25nm Pt underlayer. Both measurements were performed at a fixed temperature of 2K. The I_c(H) curve shown in this figure was generated by measuring IV curves at several applied fields and choosing a critical voltage criteria of 5 μV to extract the critical current. The V(H) curve was generated by continuously scanning the magnetic field at a junction bias current of 650μAmps. There is a clear one-to-one correspondence between flux periodicity of the two curves, although the maxima in I_c(H) are

replaced by minima in V(H), as expected. In the rest of the manuscript we will refer to the V(H) plot as the Fraunhofer plot.

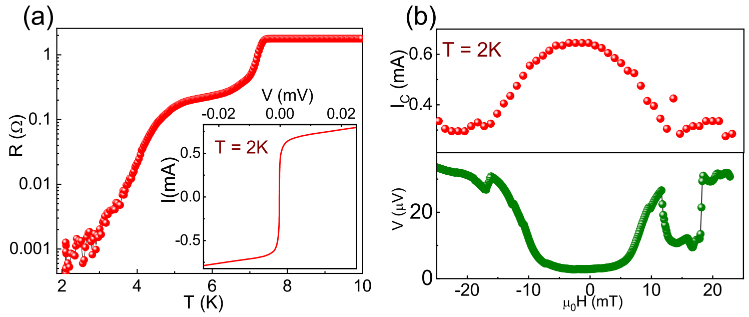

Supplementary Fig. 2: Basic characteristics of an Nb-(Pt/Cu)-Nb junction; **(a)** Resistance vs Temperature of an Nb-(Pt(25nm)/Cu)-Nb planar Josephson junction measured with a bias current of 10μA. The resistance drop at higher temperatures shows the superconducting transition of the Nb electrodes, whereas the transition near 5K corresponds to the proximatisation of the Cu barrier. The inset shows the IV curve of the same device measured at 2K. The critical current for the device was found to be 650 μAmps. Panel (b) shows the equivalence of the field dependence of critical current and junction resistance.

Supplementary note 3: Tuning Josephson junction resistance by controlling ion beam milling time

The planar Josephson junctions and SQUIDs discussed in this work were made by top-milling the Nb layer from the junction area to define the separation between the Nb electrodes. Initially, the multilayer track was narrowed down to the desired width, which was followed by top milling with a low ion current of 5pA at 30kV. After optimizing the milling time for the top Nb with thickness 150 nm, additional milling time was used to control the depth of milling into the Cu layer in an Nb-(pt/Cu)-Nb junction with 10 nm thick Pt layer, on the same chip. The schematic diagram is shown in the inset of Supplementary Fig. 3. The temperature dependent resistance of the resulting junctions are shown in the main panel of Supplementary Fig. 3. It shows that by increasing the milling time from 6.8s to 9.52s, there were clear changes in the junction resistance at 2K. The increase in the junction resistance with milling time (equivalently, the milling depth) resulted a decrease in the supercurrent and, consequently to a decrease in the quasiparticle current J_Q which determines the magnitude of the Rashba-Edelstein effect. Figure 5, in the main text shows that the offset in the Fraunhofer pattern scales with the junction resistance for the above reason.

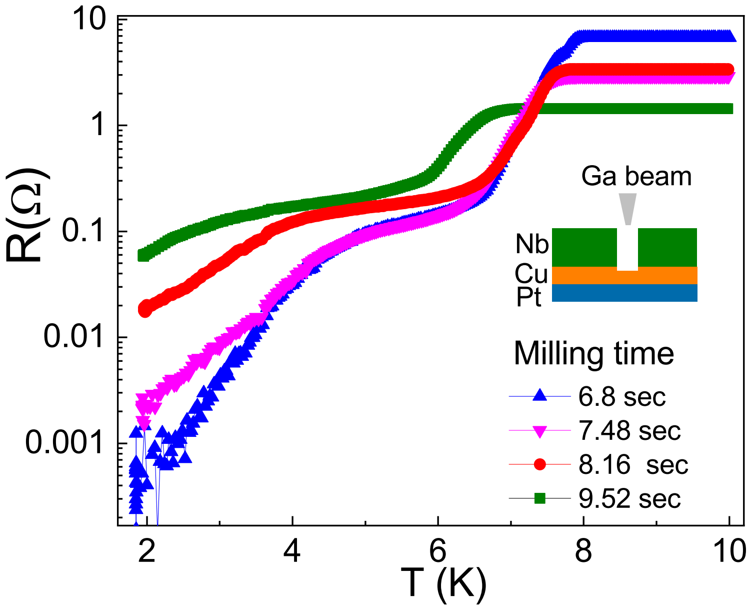

Supplementary Fig. 3: Controlling Cu thickness in the junction area by varying milling time. The R(T) plot for a series of devices fabricated on the same chip with Cu thickness variations is shown here. To control Cu thickness, the milling duration was varied as 6.8, 7.48, 8.16 and 9.52 sec. The resistance at 2K increased consistently with increasing milling time.

Supplementary note 4: Voltage response of SQUID devices with different Pt thickness

The central feature of a SQUID device is the voltage oscillation with a magnetic field around the critical current of the device. The magnetic field response of the device voltages for some representative Nb-(Pt/Cu)-Nb dc SQUIDs used in this study are plotted in Supplementary Fig. 4 for different thicknesses of the Pt layer. Clear voltage oscillations superposed on the Fraunhofer-like envelope are observed in all cases. For different thicknesses of the Pt layer, the offset ΔH between the forward and reverse Fraunhofer patterns was different, consistent with the single junction devices. Note that the SQUID oscillations on a single device did not show any change in the forward and backward scans of magnetic field, as the origin of ΔH is exclusively related to the spin moment associated with Rashba-Edelstein effect at the Pt/Cu interface under the individual Josephson Junctions.

Supplementary Fig. 5 shows voltage plots for Pt(50nm) device at different bias currents. Similar to the single junctions, the ΔH of the SQUID devices also did not show much dependence on the bias currents. As discussed in the text, the entire

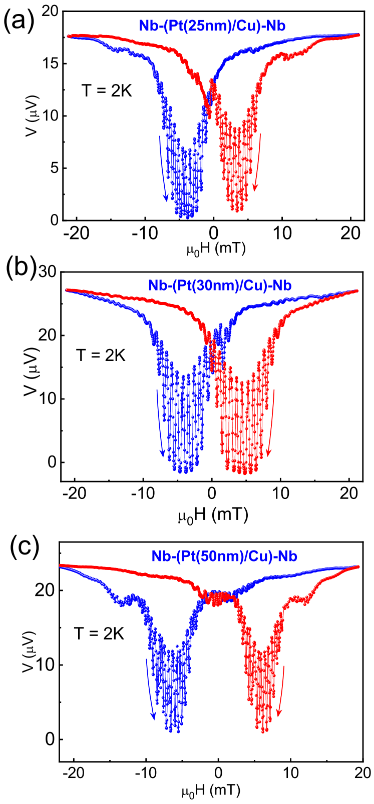

Supplementary Fig. 4: V(H) curves for SQUIDs with different Pt thicknesses. Field dependence of device resistance for the Nb-(Pt/Cu)-Nb SQUIDs with Pt thicknesses of 25, 30, and 50nm are shown in panels (a), (b), and (c), respectively. These devices were measured with their respective critical currents at 2K. All devices showed an offset between the forward and reverse sweeps of Fraunhofer patterns.

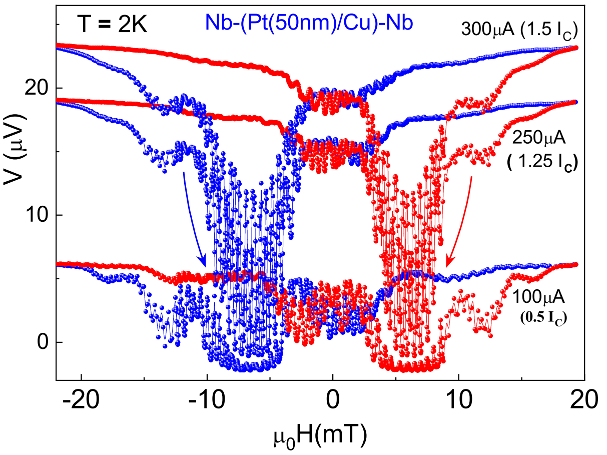

Supplementary Fig. 5: Bias current dependence of ΔH in SQUID device. Voltage response from an Nb-(Pt(50nm)/Cu)-Nb SQUID measured with bias currents equal to 100μA (0.5I$_c$), 250μA(1.25 I$_c$) and 300μA(1.5I$_c$) show the non-hysteretic shifts.

bias current is not responsible for the Rashba-Edelstein effect at the Pt/Cu interface, because, in the proximatized state of the Josephson junction most of the current is carried by the Cooper pairs. Only a fraction of the bias current J_Q present at the interface due to pair breaking effects of Pt generates the interface spin-moment. Increasing the bias current does not imply and increase in J_Q and, therefore, no change in the ΔH is observed. On the other hand, the interface J_Q changes significantly as a function of temperature and leads to a consequent change in the ΔH, as shown in Fig. 3(b) in the main text.

Supplementary note 5: Equivalence of ΔH obtained from I_c(H) and V(H) measurements

In order to verify that the observed ΔH in V(H) data is indeed equivalent to a shift of ΔH in I_c(H) data we have compared the two measurements for a single planar Nb-(Pt/Cu)-Nb junction [1]. I_C extracted from I-V measurements at various magnetic fields during the up and down sweeps of the magnetic field has been plotted in the left-hand side axis of Supplementary Fig. 6. The corresponding V(H), measured at a fixed bias current close to the critical current, has been plotted in the right-hand side axis of the same figure. We note that the magnitude of the offset between up-sweep and down-sweep data in both measurements match very well with each other, verifying the equivalence of both modes of measurements.

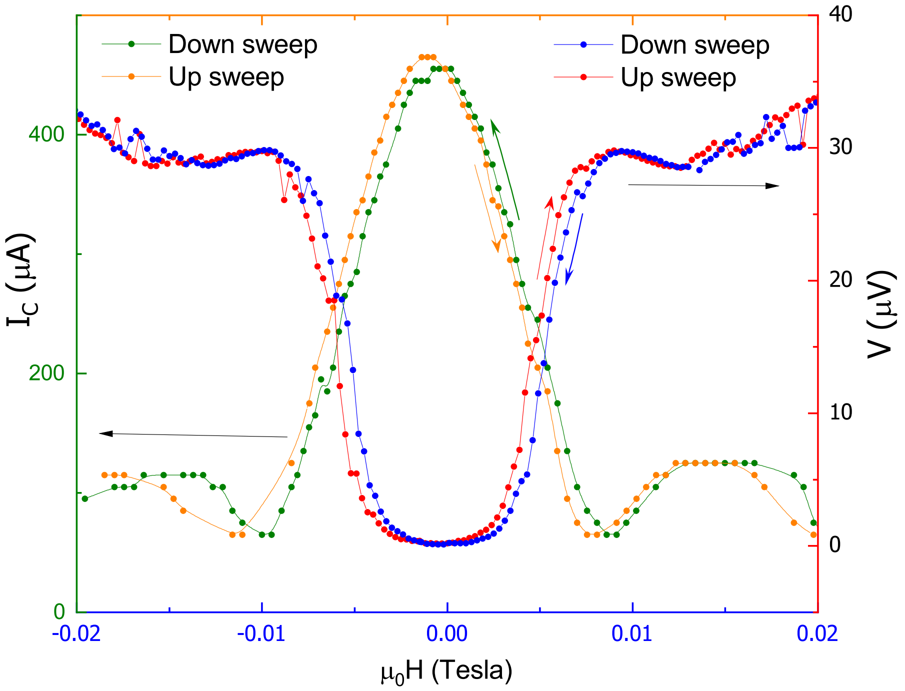

Supplementary Fig. 6: Comparison of V(H) and I_c(H) measurements: Field response of the voltage across an Nb-(Pt(12nm)/Cu)-Nb planar Josephson junction is compared with the I_c(H) response of the same junction.

Supplementary note 6: Effects of the reversed spin polarization, and changing the magnitude of the Rashba constant on ΔH

The basic architecture of our device is to position a planar Josephson junction just above a region with an in-plane non-equilibrium spin density. The applied out-of-plane (OOP) magnetic field pulls the spin-moment out of the Rashba interface, which contributes to the total flux through the junction. In this process, the spin-moment gets locked to the flux quantum and the rigidity of the flux quantum leads to the

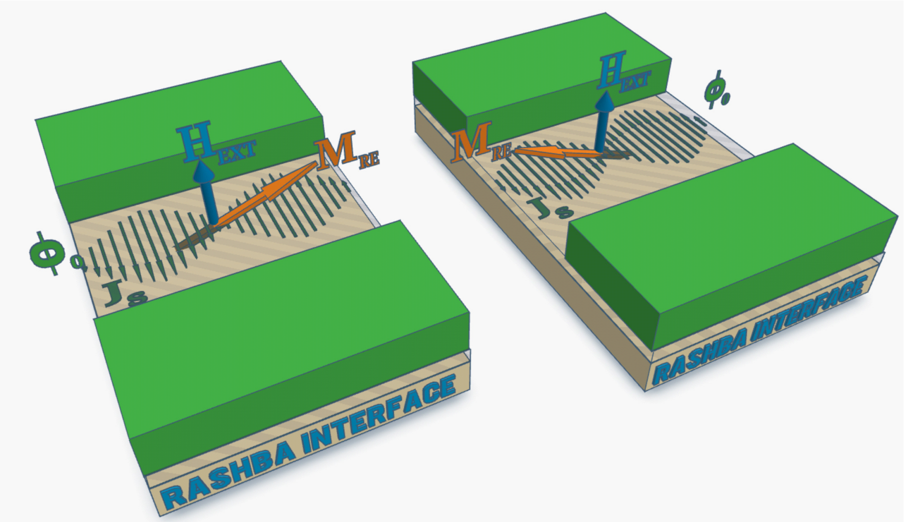

Supplementary Fig. 7: Schematic illustration of M_{RE} component: Schematics comparison of the two scenarios where the direction of Rashba-Edelstein spin polarization at the (Pt/Cu) interface has been reversed. The green arrows in the junction area indicate the critical current distribution in the proximatized area. Although the spin moment M_{RE} has a different direction in the two cases, they lock to the flux quantum in the same direction.

squeezing of the Fraunhofer pattern (Fig. 4(a) in the main text) during the down sweep of the OOP magnetic field. As a result, the junction retains a finite phase even when the external OOP magnetic field is set back to zero. In the schematic shown in Supplementary Fig. 7, we compare two scenarios where the directions of the Rashba-Edelstein spin moment M_{RE} have been reversed (either by changing stacking order or by using materials with opposite spin Hall angles in the two cases) [2]. In both cases, the spin moment locks to the flux quantum only in the direction of the applied field. Therefore, in both cases, we can expect a squeezing of the Fraunhofer pattern during the down sweep of the field only. Hence the presented technique is not sensitive to the direction of the induced non-equilibrium spin moment, but only to the magnitude of the spin-moment. To verify this argument, we have conducted the following two control experiments.

(A) Effect of reversing the direction of spin-polarization on ΔH : Under the Rashba-Edelstein model, the non-equilibrium spin expectation value can be written as

$$\frac{\langle\vec{\sigma}\rangle}{A} = \frac{\alpha_R m\hbar}{|e|\,(\alpha_R^2 m + \hbar^2\varepsilon_F)}\left[\vec{e}_z \times \vec{J}_C\right] \tag{1}$$

where $(\vec{e}_z)$ is the unit vector along the interface electric field and $(\vec{J}_C)$ is the surface current density at the Rashba interface [3]. Clearly, the direction of polarization of the induced spin-moment can be reversed either by reversing the interface electric field (changing stacking order) or by changing the direction of the interface current density. In our experimental geometry, changing the stacking order of (Pt/Cu) to (Cu/Pt) is not feasible due to the fact that the proximatization length of Ga poisoned (due to the FIB milling process) Pt is extremely small. Therefore, a planar Josephson junction with a barrier gap of 50-100 nm (gap limited by the technique of FIB milling) can not be realized by having Pt as the top layer in the barrier region. From the above expression, an alternative route to reverse the direction of spin-polarization is to change the direction of the bias current in the junction. In Supplementary Fig. 8, we compare the V(H) data of a junction, measured with positive and negative bias currents, keeping the direction of the applied magnetic field unchanged. As evident from this figure, ΔH does not suffer any change by the reversal of spin-polarization, consistent with the argument presented in the previous paragraph.

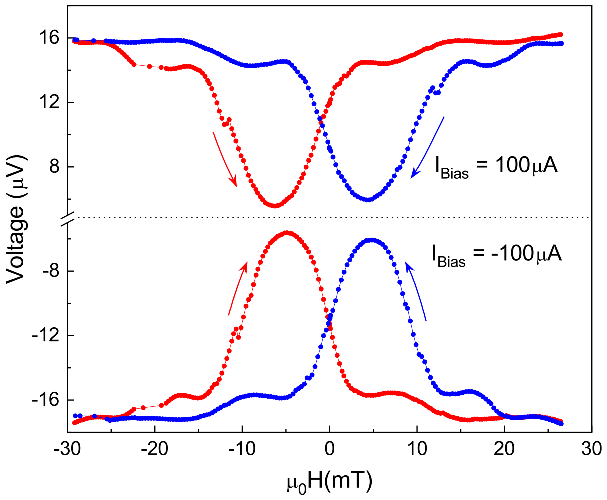

Supplementary Fig. 8: Effect of reversing spin polarization on ΔH : Comparison of the V(H) curves of a Nb-(Pt(30nm)/Cu)-Nb Josephson junction with positive and negative bias currents. The magnitude of the current was fixed at 100μA and the direction of magnetic field was kept fixed for both measurements. The offset between the up-sweep and down-sweep data remained unchanged in these measurements.

(B) Effect of spin-orbit coupling strength on ΔH: The magnitude of the spin moment generated due to the Rashba-Edelstein effect is proportional to the spin-orbit coupling strength of the heavy metal. Therefore, replacing Pt with another material is expected to change the value of ΔH in our experiment. We have performed this control experiment by replacing Pt with Nb, which has a much lower spin-orbit coupling strength. The thickness of Nb was 10 nm, which is much less than the coherence length of Nb for the film to become superconducting at 2K. We show the V(H) curve of such a junction measured at 2 K in the following figure. Compared to a junction with Pt underlayer, we find a significantly lower ΔH for the case of Nb underlayer.

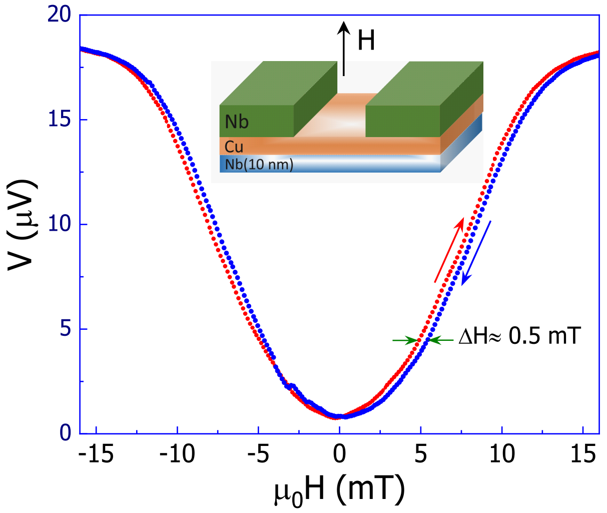

Supplementary Fig. 9: Effect of changing spin-orbit coupling strength on ΔH: Magnetic field dependence of junction voltage in a planar Josephson device where Pt was replaced with 10 nm of non-superconducting Nb. The Rashba interface, in this case, was the Nb/Cu interface. The lower spin orbit coupling strength of Nb compared to Pt leads to a weaker ΔH between the up sweep and down sweep.

Supplementary note 7: Estimation of quasiparticle current density at the Pt/Cu interface

A phase shift in the Fraunhofer pattern of a Josephson junction can occur only in presence of an additional flux. In our experiment the observed shift in the Fraunhofer patterns (H) is caused by the flux due to the spin moment M_{RE} created by the Rashba-Edelstein effect at the interface, which can be written as

$$M_{RE} = \mu_0(\Delta H/2) \tag{2}$$

The factor of 2 comes from the fact that H in the manuscript has been defined as the total shift between the up-sweep and down-sweep Fraunhofer patterns. The origin of the spin moment M_{RE} is the average non-equilibrium spin $\langle\vec{\sigma}_{RE}\rangle$. Therefore, M_{RE} can also be written as

$$\vec{M}_{RE} = g(\frac{e}{2m})\,\langle\vec{\sigma}_{RE}\rangle \tag{3}$$

Where g, e, and m are the gyromagnetic ratio, charge and mass of the electron, respectively. Therefore we can write

$$\langle\vec{\sigma}_{RE}\rangle = \mu_0\frac{\Delta H}{2}(\frac{e}{m}) \tag{4}$$

Using this expression, we can directly relate the experimentally observed parameter ΔH to the magnitude of interfacial quasiparticle current density as [4]

$$\mu_0\frac{\Delta H}{2A} = \frac{\alpha_R\hbar}{(\alpha_R^2 m + \hbar^2\varepsilon_F)}J_Q \tag{5}$$

Here A is the junction area, ε_F is the Fermi energy of Pt at the Pt/Cu interface. For the purpose of an estimation we consider the V(H) data in Supplementary Fig 8, which was measured with a bias current of 100 μ A. Using $\mu_0\Delta$H/2 = 50 Oe $\approx$ 3979 A/m, A = 50 nm $\times$ 300 nm = 1.5 $\times$ 10^{-14}m^2, ε_F = 1.4 $\times$ 10^{-18}J [5], α_R = 0.001 eV.Å $\approx$ 1.6 $\times$ 10^{-32}J.m [2], a quasiparticle surface current of 0.7 nA was estimated at the Pt/Cu interface for this particular junction. Considering the fact that the Cu layer, proximatized by the superconducting Nb electrodes, carries all the bias current, a low value of the interface quasiparticle current is very much expected. On the other hand, the direct estimation of α_R is not feasible in our case without microscopic calculations of pair breaking effects at the Pt/Cu interface, because from the measured parameters of the experiment the value of J_Q can not be estimated directly.

Supplementary References

[1] Jeon, K.-R. *et al.* Chiral antiferromagnetic josephson junctions as spin-triplet supercurrent spin valves and dc squids. *Nature Nanotechnology* 1–7 (2023).

[2] Yu, R. *et al.* Fingerprint of the inverse rashba-edelstein effect at heavy-metal/cu interfaces. *Phys. Rev. B* **102**, 144415 (2020).

[3] Johansson, A., Henk, J. & Mertig, I. Theoretical aspects of the edelstein effect for anisotropic two-dimensional electron gas and topological insulators. *Phys. Rev. B* **93**, 195440 (2016).

[4] Lee, W.-B. *et al.* Direct observation of spin accumulation and spin-orbit torque driven by rashba-edelstein effect in an inas quantum-well layer. *Physical Review B* **104**, 184412 (2021).

[5] Bordoloi, A. K. & Auluck, S. Electronic structure of platinum. *Journal of Physics F: Metal Physics* **13**, 2101 (1983). URL https://dx.doi.org/10.1088/0305-4608/13/10/019.
